# Safety and efficacy of multi-target TKI combined with nivolumab in check-point inhibitor-refractory patients with advanced NSCLC: a prospective, single-arm, two-stage study

**DOI:** 10.1186/s12885-024-12479-0

**Published:** 2024-06-11

**Authors:** Bo Zhang, Hongyu Liu, Chunlei Shi, Zhiqiang Gao, Runbo Zhong, Aiqin Gu, Tianqing Chu, Huimin Wang, Liwen Xiong, Wei Zhang, Xueyan Zhang, Bo Yan, Jiajun Teng, Weimin Wang, Hao Bai, Rong Qiao, Lei Cheng, Yanbin Kuang, Ruiying Zhao, Hua Zhong, Baohui Han

**Affiliations:** 1 Department of Respiratory and Critical Care Medicine, Shanghai Chest Hospital, Shanghai Jiao Tong University School of Medicine, Shanghai, People’s Republic of China; 2 Clinical Research Center, Shanghai Chest Hospital, Shanghai Jiao Tong University School of Medicine, Shanghai, People’s Republic of China; 3 Department of Pathology, Shanghai Chest Hospital, Shanghai Jiao Tong University School of Medicine, Shanghai, People’s Republic of China

**Keywords:** NSCLC, Checkpoint inhibitor-refractory, Nivolumab, Anlotinib

## Abstract

**Background:**

Resistance to immune checkpoint inhibitors (ICIs) represents a major unmet medical need in non-small cell lung cancer (NSCLC) patients. Vascular endothelial growth factor (VEGF) inhibition may reverse a suppressive microenvironment and recover sensitivity to subsequent ICIs.

**Methods:**

This phase Ib/IIa, single-arm study, comprised dose-finding (Part A) and expansion (Part B) cohorts. Patients with ICIs-refractory NSCLC were enrolled to receive anlotinib (a multi-target tyrosine kinase inhibitor) orally (from days 1 to 14 in a 21-day cycle) and nivolumab (360 mg every 3 weeks, intravenously) on a 21-day treatment cycle. The first 21-day treatment cycle was a safety observation period (phase Ib) followed by a phase II expansion cohort. The primary objectives were recommended phase 2 dose (RP2D, part A), safety (part B), and objective response rate (ORR, part B), respectively.

**Results:**

Between November 2020 and March 2022, 34 patients were screened, and 21 eligible patients were enrolled (6 patients in Part A). The RP2D of anlotinib is 12 mg/day orally (14 days on and 7 days off) and nivolumab (360 mg every 3 weeks). Adverse events (AEs) of any cause and treatment-related AEs (TRAEs) were reported in all treated patients. Two patients (9.5%) experienced grade 3 TRAE. No grade 4 or higher AEs were observed. Serious AEs were reported in 4 patients. Six patients experienced anlotinib interruption and 4 patients experienced nivolumab interruption due to TRAEs. ORR and disease control rate (DCR) was 19.0% and 76.2%, respectively. Median PFS and OS were 7.4 months (95% CI, 4.3-NE) and 15.2 months (95% CI, 12.1-NE), respectively.

**Conclusion:**

Our study suggests that anlotinib combined with nivolumab shows manageable safety and promising efficacy signals. Further studies are warranted.

**Trial registration:**

NCT04507906 August 11, 2020.

**Supplementary Information:**

The online version contains supplementary material available at 10.1186/s12885-024-12479-0.

## Background

Globally, lung cancer is the leading cause of cancer-related deaths with an estimated 1.76 million deaths per year [1, 2]. The advent of immune checkpoint inhibitors (ICIs) against programmed death 1 (PD-1) or programmed death ligand 1 (PD-L1) has revolutionized first-line treatment in advanced NSCLC patients without driver gene mutations. ICIs monotherapy is recommended in PD-L1 ≥ 50% [3], and ICIs in combination with chemotherapy is recommended in PD-L1 low or negative patients to boost clinical response [4–6]. However, despite potentially durable responses, most patients may experience disease progression due to ICIs resistance, which represents an urgent unmet need in subsequent treatment.

ICIs resistance is involved in a variety of mechanisms. Vascular endothelial growth factor (VEGF), a key regulator of angiogenesis, is an important treatment target in NSCLC [7]. Recent studies have suggested that VEGF is also associated with immune suppression. VEGF can suppress the maturation of dendritic cells, thus interfering with T cell priming [8]. In addition, VEGF-A induces the thymocyte selection-associated high mobility group box protein (TOX)-mediated exhaustion of CD8 + T cells via transcriptional reprogramming [9]. Meanwhile, Treg cells secrete VEGF that promote vascular immaturity, impairing the penetration of CD8 + T cell [10]. Last, VEGF hinders lymphocytes mobilization and across of the endothelial cells through its interaction with Fas ligand [11, 12]. These results led to the expectation that simultaneous targeting immunity and tumor vessels may normalize aberrant vascular-immune crosstalk, reverse the suppressive microenvironment and recover sensitivity to subsequent ICIs.

Anlotinib is a multitarget tyrosine kinase inhibitor (TKI). The targets of anlotinib include VEGFR, fibroblast growth factor receptor (FGFR), platelet-derived growth factor receptor (PDGFR) and cKit [13]. In our previous phase Ib study, the combination of anlotinib with sintilimab, a fully humanized PD-1 monoclonal antibody, showed a potent synergetic effect in treatment-naïve advanced NSCLC patients without driver gene alterations [14]. The combination conferred an objective response rate (ORR) of 72.7% and a median progression-free survival (PFS) of 15 months [14].

In this phase Ib/IIa study, we investigated the recommended phase 2 dose (RP2D), safety and antitumor activity of this combination in ICIs-refractory advanced NSCLC patients.

## Method

### Study design and objective

This phase Ib/IIa, open-label, single-center study comprised dose-finding (Part A) and expansion (Part B) cohorts. The primary objectives were RP2D (part A), safety (part B), and ORR (part B). The secondary aim of part B included disease control rate (DCR; ORR and stable disease rate), duration of response (DOR; from the first radiographic documentation of clinical response to first disease progression or death of any cause), PFS (from treatment initiation to the first radiographic disease progression or death of any cause), and overall survival (OS; from treatment initiation to all-cause death). This study has been registered at ClinicalTrials.gov (NCT04507906).

### Patients

Patients aged 18–75 years, regardless of PD-L1 expression, were eligible for enrollment if they had pathologically- or cytologically-confirmed locally advanced or metastatic NSCLC and an Eastern Cooperative Oncology Group Performance Status (ECOG PS) score of 0–1. Prior treatment with ICIs against PD-1 and PD-L1 was allowed. Patients had to be sequentially or concurrently treated with chemotherapy, or ineligible for chemotherapy. Palliative radiotherapy had to be completed 7 days before the first dose of study drugs. Measurable disease was also required, and asymptomatic brain metastasis was allowed. Patients who do not have available targeted therapy (e.g. HER-2, KRAS) were eligible. Patients who discontinued ICIs due to adverse events, those who previously received anti-angiogenesis treatment, those with active central nervous system (CNS) metastases or obvious hemorrhage symptoms, patients with active autoimmune disease or showed primary resistance (defined as a clinical scenario where a cancer does not respond to an immunotherapy strategy after 6 weeks exposure of immunotherapy) [15] were excluded. Full eligibility criteria can be found in the study protocol.

Qualitative immunohistochemistry (IHC) method was used to detect PD-L1 protein expression in formalin-fixed, paraffin-embedded (FFPE) NSCLC tissues. PD-L1 IHC 22C3 pharmDx (Dako North America, Inc, California, USA) with monoclonal mouse anti-PD-L1 (clone 22C3) antibody was performed using EnVision FLEX visualization system on Autostainer Link 48 (Dako North America, Inc, California, USA). PD-L1 protein expression was determined by means of the Tumor Proportion Score (TPS), which shows the percentage of viable tumor cells with partial or complete membrane staining.

### Study procedures and treatment

The study was approved by the ethics committee of Shanghai Chest Hospital (LS2025), and all patients provided written informed consent. This study was performed according to the guidelines for Good Clinical Practice and the Declaration of Helsinki.

#### Part A: Combination dose finding

A “3 + 3”design was used in the dose finding cohort. Dosing started at the full dose of both drugs (anlotinib: 12 mg/day orally, 14 days on and 7 days off; nivolumab: 360 mg every 3 weeks) due to the well-established and non-overlapping safety profiles of nivolumab and anlotinib, and the desire to treat patients at effective dose levels. Two dose de-escalation steps were included: dose level 2 (anlotinib: 10 mg/day orally, 14 days on and 7 days off; nivolumab: 360 mg every 3 weeks) and dose level 3 (anlotinib 8 mg/day orally, 14 days on and 7 days off; nivolumab: 360 mg every 3 weeks). The determination of dose-limiting toxicity (DLTs) was made by investigators after safety data from each dose level had been reviewed.

#### Part B: Expansion cohort

If RP2D was reached in part A, eligible patients were enrolled in part B and received anlotinib plus nivolumab (anlotinib RP2D: 14 days on and 7 days off; nivolumab: 360 mg every 3 weeks) until disease progression, withdrawal of consent, or unacceptable toxicity. Continued treatment after disease progression was allowed if the treating physician identified clinical benefit. Tumor assessment was performed at baseline and every 6 weeks according to response evaluation criteria in solid tumors (RECIST) version 1.1. Adverse events (AEs) were reported based on the National Cancer Institute Common Terminology Criteria for Adverse Events, version 4.03.

### Statistical methods

The primary endpoint of the study was ORR as determined by investigator review. Sample size was calculated by a Simon’s two-stage design method based on the following parameters: α = 0.1, 1-β = 0.8, P0 = 0.05, and P1 = 0.15. The optimal two-stage design was used to test the null hypothesis that *P* ≤ 0.05 versus the alternative that *P* ≥ 0.15. After testing the drug in 20 patients at the first stage, the trial was planned to be terminated if there was ≤ 1 response. If the trial proceeded on to the second stage, it was planned to assess a total of 56 patients. If the total number of respondents was ≤ 4, the method was considered to be noneffective. Assuming a dropout rate of 5%, the study planned to enrol 62 patients.

Efficacy and safety analyses are presented based on those patients who received at least 1 dose of the study drugs. Non-evaluable patients (i.e. those who dropped out before the first radiological assessment) were included in the denominator when calculating ORR. Median PFS and OS are presented by Kaplan–Meier curves. All data were analyzed using SPSS 23.

### Exploratory biomarker analyses

#### Biomarker analyses

Blood biopsies were collected at baseline, first assessment, and at disease progression. These samples were then subjected to next-generation sequencing, as described previously [16, 17]. Briefly, circulating cell-free DNA (cfDNA) was extracted using a QIAamp Circulating Nucleic Acid kit per manufacturer’s instructions (Qiagen, Hilden, Germany). The extracted DNA was subsequently sheared, and fragments between 200–400 bp were purified (Agencourt AMPure XP Kit, Beckman Coulter, CA, USA), hybridized with capture probes baits, selected, amplified, and subjected to targeted capture. The size and quality of the fragments were assessed with a Bioanalyzer 2100 (Agilent Technologies, CA, USA). The indexed samples were sequenced on Nextseq 500 (Illumina, Inc., CA, USA) with paired-end reads and an average sequencing depth of 1000 × for tissue and 10,000 × for liquid biopsy samples. Sequence data were processed with a bioinformatic pipeline, as reported previously [18].

#### Blood tumor mutation burden (bTMB) calculation

bTMB was computed as the ratio between the total number of non-synonymous mutations detected and the total coding region size of the targeted panel. Only mutations with allelic fraction (VAF) of ≥ 0.2% were included. Also, the maximum allelic fraction (MSAF) of the corresponding sample was required to be ≥ 0.5%.

#### Blood-based intratumor heterogeneity (bITH) calculation

bITH is an index of intertumoral heterogeneity based on genomic profiles acquired from blood biopsies. The bITH score was calculated as described previously [19]. MSAF-corrected VAFs (MCVs) were first calculated by dividing VAF by MSAF. bITH was then calculated using the following equation:

$$\text{bITH}= -\sum_{i=1}^{n}{\varphi }_{i}\bullet {P}_{i}\bullet \text{ln}({P}_{i})$$

where n is the number of bins (default 10), ${P}_{i}$ is the probability of MCVs located in respective bins, and ${\varphi }_{i}$ is the average of the corresponding MCV bin endpoints.

## Results

### Baseline characteristics

**Fig. 1:**
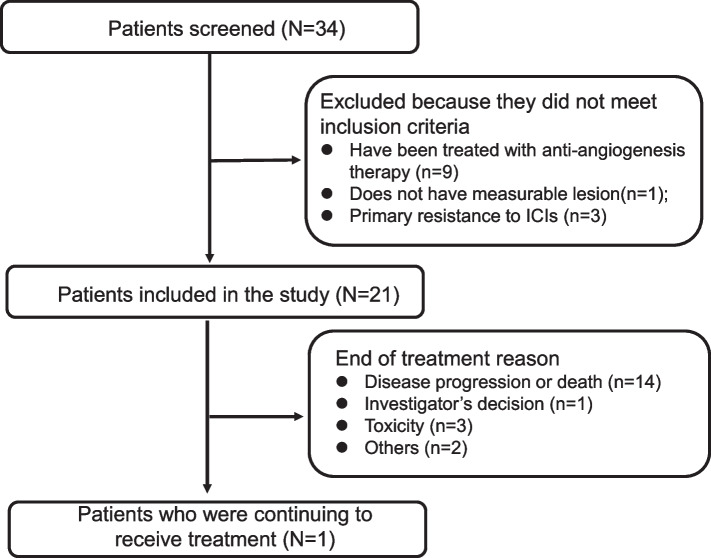
Study flowchart

This study was terminated early due to the breakout of coronavirus disease 2019, so data should be considered as exploratory. Between November 2020 and March 2022, 34 patients were screened and 21 eligible patients were enrolled, including 6 patients enrolled in part A (Fig. 1). Baseline characteristics are listed in Table 1. The median patient age was 65 years, 47.6% had adenocarcinoma, 47.6% were smokers, and 85.7% were male. All patients received PD-1 as previous treatment, including pembrolizumab (eight patients), penpulimab (four patients), sintilimab (one patient), camrelizumab (two patients), toripalimab (three patients), tislelizumab (two patients), and HX-008 (one patient). Two cases were administered with pembrolizumab monotherapy before participating in this trial. Brain metastasis at baseline was present in 9.5% of patients. 5 patients (23.8%) had driver mutation (KRAS G12C: one patient; HER-2 insertion: three patients; BRAF K601E: one patient). Patients had received a median of one previous lines of treatment. The median PFS was 8.5 months (95% CI 5.4–17.0 m) of frontline ICIs treatment (Figure S1).

**Table 1:** Baseline characteristics

Characteristics (*n* = 21)	No	%
**Median age (years, range)**	65 (34–75)	
**Sex**		
Male	18	85.7
Female	3	14.3
**ECOG**		
0	5	23.8
1	16	76.2
**Smoking status**		
Yes	10	47.6
No	11	52.4
**Brain metastasis**		
Present	2	9.5
Absent	19	90.5
**Histology**		
Adenocarcinoma	10	47.6
Squamous	10	47.6
Others	1	4.8
**PD-L1 expression**		
≥ 1%	6	28.6
< 1%	6	28.6
Unknown	9	42.8
**Previous treatment line**		
1	15	71.4
2	4	19.0
3	2	9.6
**Driver genes mutation**		
No	16	76.2
Yes	5	23.8
**Types ICIs**		
PD-1	21	100
PD-L1	0	0
**Chemotherapy**		
Yes	19	90.5
No	2	9.5

### Determination of RP2D and efficacy

In part A, 1 of the first 3 patients experienced DLT (grade 3 proteinuria); thus, an additional 3 patients were enrolled. These 3 patients did not experience DLT, and the RP2D was determined as anlotinib (12 mg/day orally, 14 days on and 7 days off) and nivolumab (360 mg every 3 weeks intravenously).

**Fig. 2:**
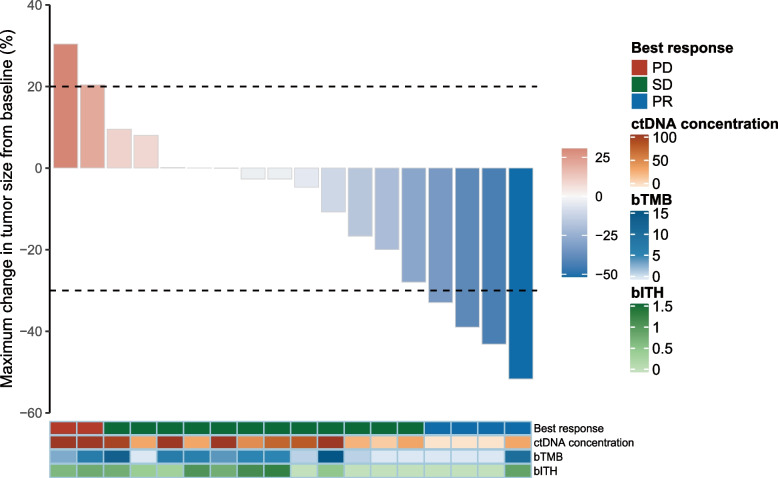
Maximum change in target lesion

**Fig. 3:**
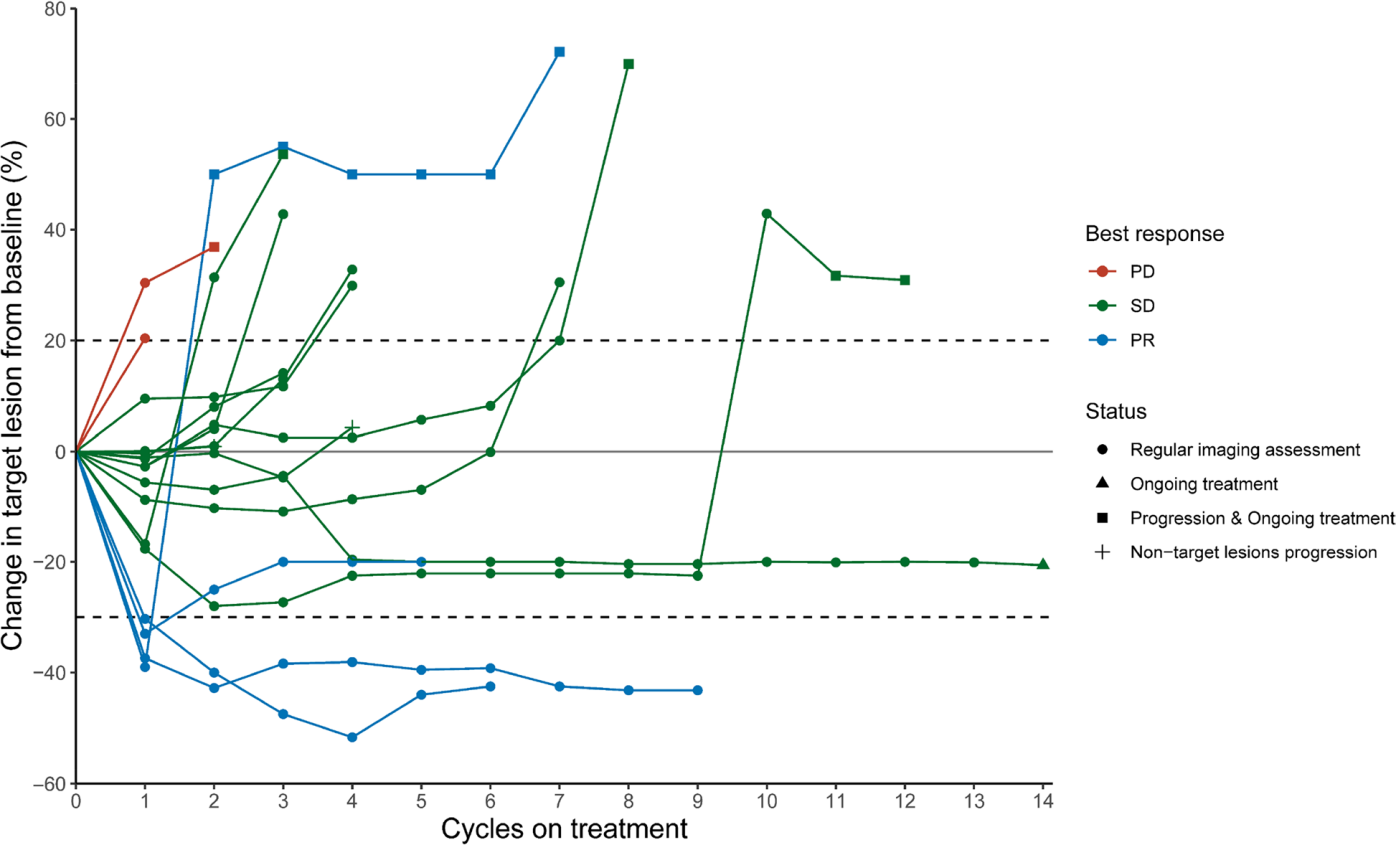
Longitudinal change in sum of longest target lesion diameters from baseline

Among the 21 patients, 4 patients had a confirmed partial response, and ORR was 19.0%. No complete responses were observed. An additional 12 patients were assessed as stable disease and DCR was 76.2%, with 4 patients showing tumor shrinkage of more than 10% (Fig. 2). Three patients were not evaluable for response before the first radiological assessment (1 for DLT and discontinued treatment, 1 for withdrawal of consent, and 1 for fracture). The median response depth was − 3.7%. Among the 4 patients who had a clinical response, 1 patient showed ongoing response at the cut-off date (21, August 2023; median follow-up time: 22.2 months, 95% confidence interval (CI) 19.5 m-not evaluable), with the other 3 patients discontinuing treatment (progressive disease: one patient; safety: one patient; death: one patient) (Fig. 3).

**Fig. 4:**
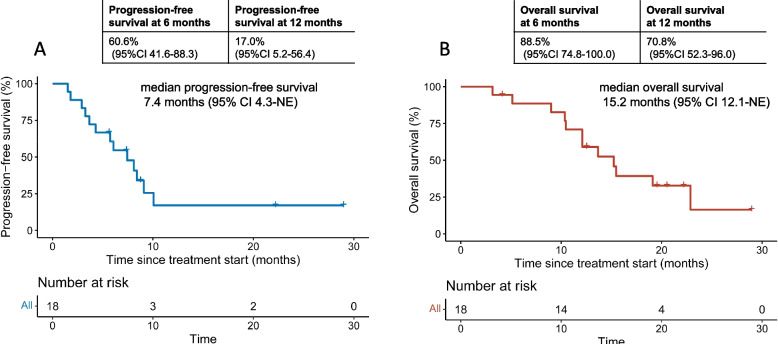
Median progression-free survival (**A**) and overall survival (**B**)

The median follow-up time was 22.2 months (95% CI 19.5 m-not evaluable). At the time of analysis, 18 PFS events had been reported, and the median PFS was 7.4 months (95% CI, 4.3 m-not evaluable). The 6- and 12-month PFS rates were 60.6% (95%CI 41.6–88.3) and 17.0% (95% CI 5.2–56.4), respectively (Fig. 4A). A total of 11 patients initiated subsequent treatment, including chemotherapy (6 patients), TKI (4 patients), anti-angiogenesis therapy (5 patients), another PD-1 or PD-L1 inhibitor (2 patients), and other treatment (1 patient). 14 deaths were reported, and median OS was 15.2 months (95% CI, 12.1- not evaluable). The 6- and 12-month OS rates were 88.5% (95% CI 74.8–100.0) and 70.8% (95% CI 52.3–96.0), respectively (Fig. 4B).

High PD-L1 expression (≥ 50%) was detected in three patients, two patients had PR (-43.2% and -52.7%). PFS were 14.3 m (censored) and 8.4 m, respectively. One patient was assessed as stable disease (+ 8%) and PFS was 4.3 m.

8 patients received pembrolizumab-based ICI treatment (2 monotherapy). There were 2 patients and 4 patients were assessed as PR and SD respectively. One patient had PD and not evaluable. ORR and DCR in these patients were 25.0% and 75.0%.

### Safety

Twenty-one patients who had received at least 1 dose of treatment drugs were included in the safety analysis. AEs of any cause and treatment-related AEs (TRAEs) were reported in all patients during treatment. The most common TRAEs included thyroid dysfunction (*n* = 8/38.1%, grade 1: *n* = 6/28.6%, grade 2: *n* = 2/9.5%), fatigue (*n* = 8/38.1%, all grade 1), bleeding (*n* = 7/33.3%, grade 1: *n* = 5/23.8%, grade 2: *n* = 2/9.5%), proteinuria (*n* = 6/28.6%, grade 1: *n* = 4/19.0%, grade 2: *n* = 1/4.8%, grade 3: *n* = 1/4.8%), pain (*n* = 6/28.6%, grade 1: *n* = 3/14.3%, grade 2: *n* = 3/14.3%), elevated aspartate aminotransferase (*n* = 4/19.0%, all grade 1), platelet decrease (*n* = 4/19.0%, grade 1: *n* = 3/14.3%, grade 2: *n* = 1/4.8%), rash (*n* = 4/19.0%, grade 1: *n* = 2/9.5%, grade 2: *n* = 2/9.5%), and elevated alanine aminotransferase in 3 patients (*n* = 3/14.3%, all grade 1). Two patients (9.5%) experienced grade 3 TRAEs (proteinuria and bronchial fistula). No grade 4 or higher AEs were observed. Serious AEs were reported in 4 patients. Overall, 6 and 4 patients experienced anlotinib and nivolumab discontinuation, respectively, due to TRAEs. Dose reduction to anlotinib 10 mg at any time was required in 5 patients, and no patients decreased to anlotinib 8 mg. Safety data are summarized in Table 2.

**Table 2:** Summary of adverse events

Adverse Events *N *= 21	Any grade	Grade 1	Grade 2	Any grade TRAE	Grade 1 TRAE	Grade 2 TRAE
Fatigue	8 (38.1%)	8 (38.1%)	0	8 (38.1%)	8 (38.1%)	0
Thyroid dysfunction	8 (38.1%)	6 (28.6%)	2 (9.5%)	8 (38.1%)	6 (28.6%)	2 (9.5%)
Bleeding	7 (33.3%)	5 (23.8%)	2 (9.5%)	7 (33.3%)	5 (23.8%)	2 (9.5%)
proteinuria	6 (28.6%)	4 (19.0%)	1(4.8%)	6 (28.6%)	4 (19.0%)	1(4.8%)
Pain	6(28.6%)	3 (14.3%)	3 (14.3%)	5 (23.8%)	3 (14.3%)	2 (9.5%)
Elevated AST	4 (19.0%)	4 (19.0%)	0	4 (19.0%)	4 (19.0%)	0
Decreased platelet count	4 (19.0%)	3 (14.3%)	1(4.8%)	4 (19.0%)	3 (14.3%)	1(4.8%)
rash	4 (19.0%)	2 (9.5%)	2 (9.5%)	4 (19.0%)	2 (9.5%)	2 (9.5%)
Elevated ALT	3 (14.3%)	3 (14.3%)	0	3 (14.3%)	3 (14.3%)	0
Elevated blood glucose	3 (14.3%)	3 (14.3%)	0	2 (9.5%)	2 (9.5%)	0
hoarseness	3 (14.3%)	3 (14.3%)	0	0	0	0
hyponatremia	2 (9.5%)	2 (9.5%)	0	2 (9.5%)	2(9.5%)	0
Prolongation of QTc interval	2 (9.5%)	2 (9.5%)	0	2 (9.5%)	2(9.5%)	0
hypochloremia	2 (9.5%))	2 (9.5%)	0	2 (9.5%))	2(9.5%)	0
anemia	2 (9.5%)	2 (9.5%)	0	2 (9.5%)	2(9.5%)	0
Hand-foot syndrome	2 (9.5%)	1(4.8%)	1(4.8%)	2 (9.5%)	1(4.8%)	1(4.8%)
hypertension	2 (9.5%)	1(4.8%)	1(4.8%)	2 (9.5%)	1(4.8%)	1(4.8%)
pneumonia	2 (9.5%)	0	2 (9.5%)	2 (9.5%)	0	2 (9.5%)
mucositis	2 (9.5%)	0	2 (9.5%)	2 (9.5%)	0	2 (9.5%)
hypomagnesemia	1 (4.8%)	1(4.8%)	0	1 (4.8%)	1(4.8%)	0
Elevated amylase	1(4.8%)	1(4.8%)	0	1(4.8%)	1(4.8%)	0
Elevated alkaline phosphatase	1(4.8%)	1(4.8%)	0	1(4.8%)	1(4.8%)	0
dizziness	1(4.8%)	1(4.8%)	0	1(4.8%)	1(4.8%)	0
urinary tract infection	1(4.8%)	1(4.8%)	0	1(4.8%)	1(4.8%)	0
cough	1(4.8%)	1(4.8%)	0	0	0	0
Decreased white blood cell count	1(4.8%)	1(4.8%)	0	1(4.8%)	1(4.8%)	0
hypokalemia	1(4.8%)	1(4.8%)	0	1(4.8%)	1(4.8%)	0
shingles	1(4.8%)	0	1(4.8%)	0	0	1(4.8%)
bronchial fistula	1(4.8%)	0	0	1(4.8%)	0	1(4.8%)
Decreased neutrophil count	1(4.8%)	0	1(4.8%)	1(4.8%)	0	1(4.8%)

### Exploratory biomarker analyses

Using blood samples collected at baseline, we conducted several exploratory analyses to evaluate genomic abnormalities, bTMB, ctDNA, and bITH for predicting clinical outcomes in patients receiving nivolumab plus anlotinib. Patients with lower ctDNA concentration (MSAF < 5%) had longer OS (hazard ratio (HR) 0.16, 95% CI 0.04–0.59, *P* = 0.002; Figure S2A) than those with higher levels (MSAF ≥ 5%), in which a similar trend was also observed in PFS (HR 0.35, 95% CI 0.11–1.11, *P* = 0.064; Figure S2B), although not statistically significant. PFS was not significantly associated with bITH (HR = 0.64, 95%CI 0.21–1.97, *P* = 0.43, Figure S3A) while for bITH-low patients, the OS was better than that for bITH-high patients at the baseline (HR 0.38, 95% CI 0.11–1.32, *P* = 0.11, Figure S3B). TP53 alterations were the most common co-mutation (12/18) (Figure S4). OS (*P* = 0.32; Figure S5A) and PFS (*P* = 0.47; Figure S5B) were not significantly different between bTMB-low (cut-off at cohort median) vs. bTMB-high.

## Discussion

In this prospective phase I/II study, full-dose anlotinib combined with nivolumab was tolerable and did not show unexpected safety signals. The most common side effects were grade 1 or 2 and manageable with supportive care. In addition, this combination showed encouraging antitumor activity in patients with ICIs-refractory advanced NSCLC.

In this study, all patients experienced TRAEs, but most of them were grade 1 or 2, and these events were resolved or recovered via supportive care or treatment delay. In addition, most treatment-related serious AEs were grade 3 or lower and manageable. In our study, hypothyroidism was the most common AE, possibly due to the overlapping side effects of anlotinib and nivolumab [13, 20]. The frequencies of all grade-specific AEs judged more likely to be associated with antiangiogenic treatment, such as proteinuria, were similar to results reported previously [21]. On the contrary, the occurrence of serious specific AEs identified as potential effects of immunotherapy, such as pneumonitis, was lower than in previously published data [3], which is mainly attributed to the exclusion of patients who had experienced serious immune-related side effects. In a phase I/II study, 67% patients experienced grade 3–4 TRAEs when treated with lenvatinib combined with pembrolizumab, even when the lenvatinib dose was decreased from 24 mg/day to 20 mg/day [22]. In another phase II study, a combination of camrelizumab plus famitinib resulted in grade 3 or higher AEs in 65.8% of patients [23]. The favorable safety profile of the combination studied in our study allowed for full-dose treatment with both agents.

Chemotherapy is the standard treatment option in ICIs-refractory NSCLC but shows limited efficacy. For these patients, there is a largely unmet medical need for a chemotherapy-free option with favorable anti-tumor activity. In our study, an objective response was observed in 19% of patients, which represents a trend for benefit. In a single arm study, sitravatinib with nivolumab didn’t meet its primary endpoint [24]. However, in this study, 28.2% (35/124) ICIs-experienced patients had no prior clinical benefit [24]. Our results were supported by a randomized study. Reckamp et al. reported that 22% of NSCLC patients who are refractory to ICIs may respond to ramucirumab combined with pembrolizumab [25]. These results were also confirmed by a similarly-designed study in which about 12.5% of patients had a clinical response to atezolizumab plus bevacizumab [26]. Collectively, these clinical results provide important support for this combination.

In addition to efficacy and toxicities, we also conducted exploratory analyses to identify predictors of clinical outcomes in patients receiving nivolumab and anlotinib. In our study, the higher level of ctDNA concentration showed significantly worse OS but not PFS. In addition, while no significant differences in survival outcomes were observed among patients with different TP53 status or levels of bITH and bTMB, patients with a lower level of bITH demonstrated a tendency of better OS than that with a higher level of bITH. A further investigation with a larger sample size is warranted.

Our study is limited by its small sample size and the fact that all participants are from China. The safety and efficacy of this combination in NSCLC patients of other ethnicities remain to be confirmed. In addition, this is a single-arm study that lacks a control group, so data should be interpreted with caution.

In summary, our study suggests that full-dose anlotinib combined with nivolumab shows manageable safety and promising efficacy signals. The findings need to be confirmed in further studies with an expanded sample size.

### Supplementary Information

Supplementary Material 1: Figure S1. Median PFS of frontline ICIs treatment. Figure S2. Kaplan-Meier survival curves showing (A) the overall survival and (B) progression-free survival of baseline ctDNA -high or -low patients. Cut-off was set at 5% of MSAF. Figure S3. Kaplan-Meier survival curves showing (A) the progression-free survival and (B) overall survival of bITH-high or -low patients. Cut-off was set at cohort median. Figure S4. Oncoplot exhibiting the top 20 genomic mutations of baseline plasma samples from 18 advanced NSCLC patients. The top histogram depicting mutation counts of the top 20 genes per sample, and the right histogram showing the mutations counts of corresponding genes in the 18 patients. Figure S5. Kaplan-Meier survival curves showing (A) the overall survival and (B) progression-free survival of bTMB-high or -low patients. Cut-off was set at cohort median.

## Data Availability

Data from this study are available upon reasonable request to the corresponding author.

## Abbreviations

TKI: Tyrosine kinase inhibitor
ICI: Immune checkpoint inhibitors
NSCLC: Non-small cell lung cancer
VEGF: Vascular endothelial growth factor
RP2D: Recommended phase 2 dose
TOX: Thymocyte selection-associated high mobility group box protein
AE: Adverse event
TRAE: Treatment-related adverse event
ORR: Objective response rate
DCR: Disease control rate
PFS: Progression-free survival
OS: Overall survival
CI: Confidence interval
PD-1: Programmed death 1
FGFR: Fibroblast growth factor receptor
PDGFR: Platelet-derived growth factor receptor
DOR: Duration of response
ECOG PS: Eastern Cooperative Oncology Group Performance Status
CNS: Central nervous system
IHC: Immunohistochemistry
FFPE: Formalin-fixed, paraffin-embedded
TPS: Tumor proportion score
cfDNA: Cell-free DNA
bTMB: Blood tumor mutation burden
bITH: Blood-based intratumor heterogeneity
DLT: Dose limiting toxicity
RECIST: Response evaluation criteria in solid tumors
HR: Hazard ratio
COVID-19: Coronavirus disease 2019
