# Supplementary material for: Safety and efficacy of multi-target TKI combined with nivolumab in check-point inhibitor-refractory patients with advanced NSCLC: a prospective, single-arm, two-stage study
Source: BMC Cancer. 2024 Jun 11;24:715. doi: 10.1186/s12885-024-12479-0 (PMC11165816; doi:10.1186/s12885-024-12479-0)
Supplement: Supplementary file 1 — Supplementary Material 1: Figure S1. Median PFS of frontline ICIs treatment. Figure S2. Kaplan-Meier survival curves showing (A) the overall survival and (B) progression-free survival of baseline ctDNA -high or -low patients. Cut-off was set at 5% of MSAF. Figure S3. Kaplan-Meier survival curves showing (A) the progression-free survival and (B) overall survival of bITH-high or -low patients. Cut-off was set at cohort median. Figure S4. Oncoplot exhibiting the top 20 genomic mutations of baseline plasma samples from 18 advanced NSCLC patients. The top histogram depicting mutation counts of the top 20 genes per sample, and the right histogram showing the mutations counts of corresponding genes in the 18 patients. Figure S5. Kaplan-Meier survival curves showing (A) the overall survival and (B) progression-free survival of bTMB-high or -low patients. Cut-off was set at cohort median. [file 12885_2024_12479_MOESM1_ESM.pdf]

Figure S1

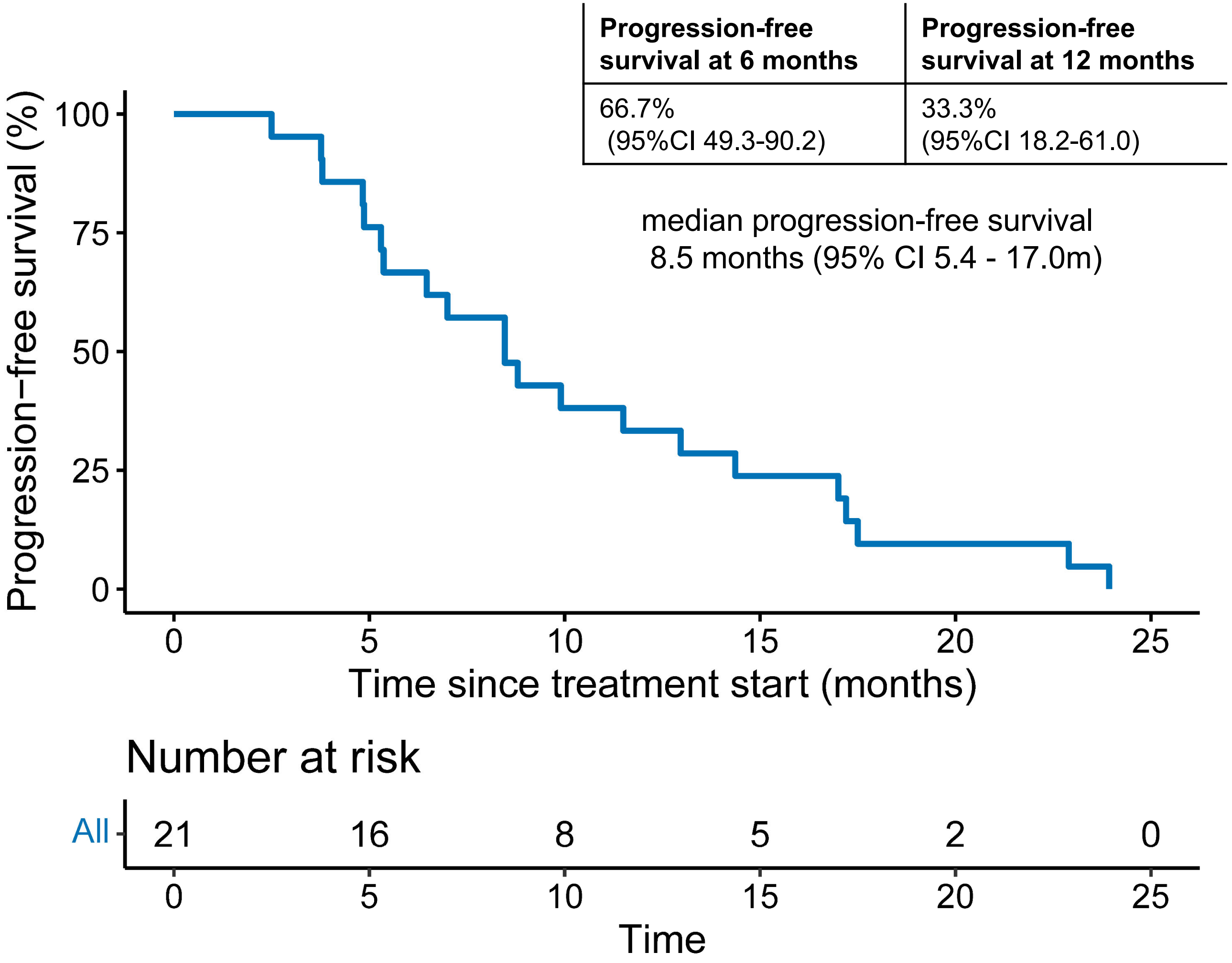
Progression-free survival at 6 months
66.7%
(95%CI 49.3-90.2)
Progression-free survival at 12 months
33.3%
(95%CI 18.2-61.0)
median progression-free survival
8.5 months (95% CI 5.4 - 17.0m)
Progression−free survival (%)
100
75
50
25
0
0
5
10
15
20
25
Time since treatment start (months)
Number at risk
All
21
16
8
5
2
0
0
5
10
15
20
25
Time

Figure S2

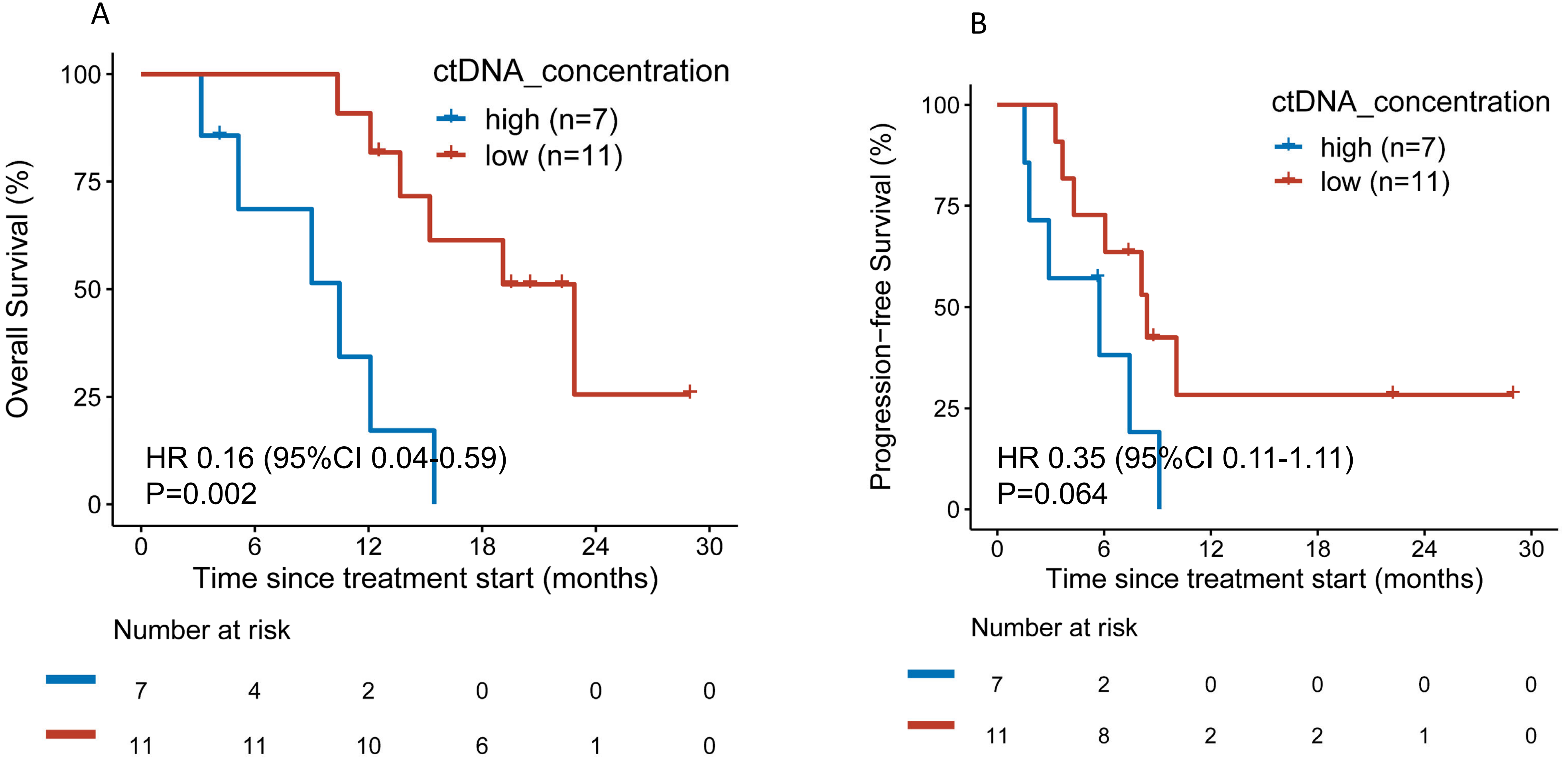
A
ctDNA_concentration
high (n=7)
low (n=11)
Overall Survival (%)
100
75
50
25
0
HR 0.16 (95%CI 0.04-0.59)
P=0.002
0 6 12 18 24 30
Time since treatment start (months)
Number at risk
7 4 2 0 0 0
11 11 10 6 1 0
B
ctDNA_concentration
high (n=7)
low (n=11)
Progression−free Survival (%)
100
75
50
25
0
HR 0.35 (95%CI 0.11-1.11)
P=0.064
0 6 12 18 24 30
Time since treatment start (months)
Number at risk
7 2 0 0 0 0
11 8 2 2 1 0

Figure S3

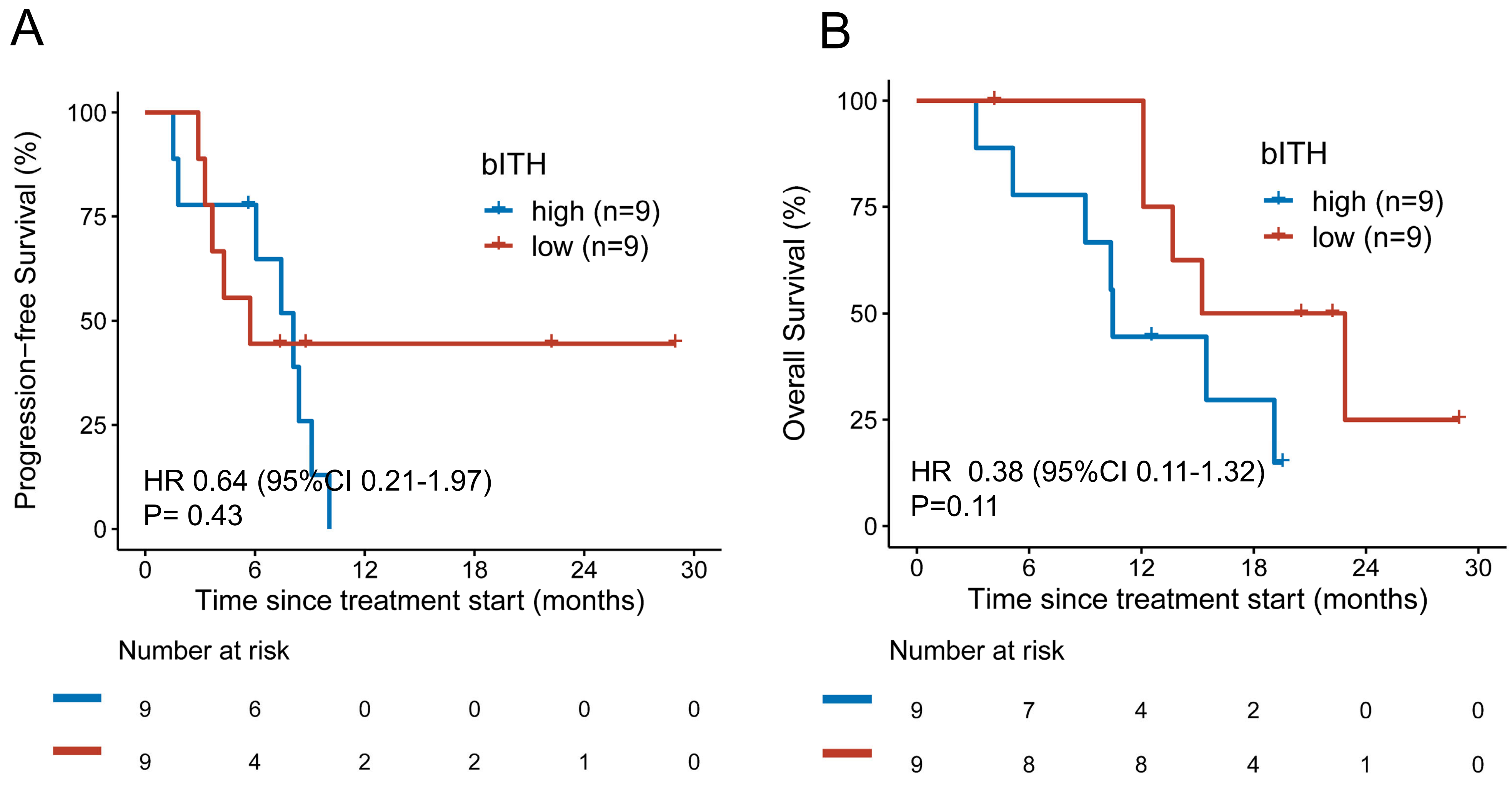
A
100
75
50
25
0
Progression−free Survival (%)
bITH
high (n=9)
low (n=9)
HR 0.64 (95%CI 0.21-1.97)
P= 0.43
0
6
12
18
24
30
Time since treatment start (months)
Number at risk
9 6 0 0 0 0
9 4 2 2 1 0
B
100
75
50
25
0
Overall Survival (%)
bITH
high (n=9)
low (n=9)
HR 0.38 (95%CI 0.11-1.32)
P=0.11
0
6
12
18
24
30
Time since treatment start (months)
Number at risk
9 7 4 2 0 0
9 8 8 4 1 0

Figure S4

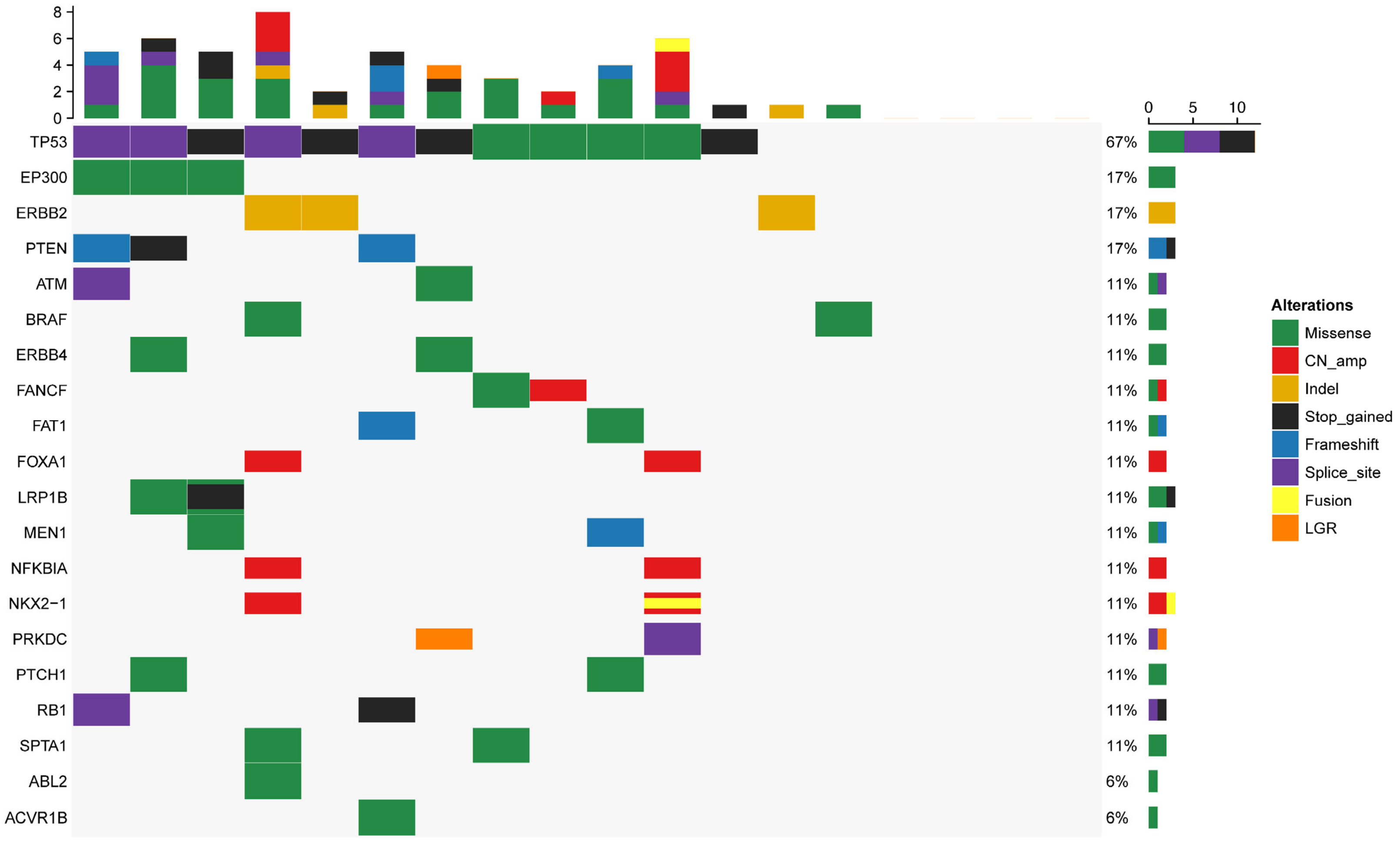

8
6
4
2
0
TP53
EP300
ERBB2
PTEN
ATM
BRAF
ERBB4
FANCF
FAT1
FOXA1
LRP1B
MEN1
NFKBIA
NKX2−1
PRKDC
PTCH1
RB1
SPTA1
ABL2
ACVR1B
67%
17%
17%
17%
11%
11%
11%
11%
11%
11%
11%
11%
11%
11%
11%
11%
11%
11%
6%
6%
0
5
10
Alterations
Missense
CN_amp
Indel
Stop_gained
Frameshift
Splice_site
Fusion
LGR

Figure S5

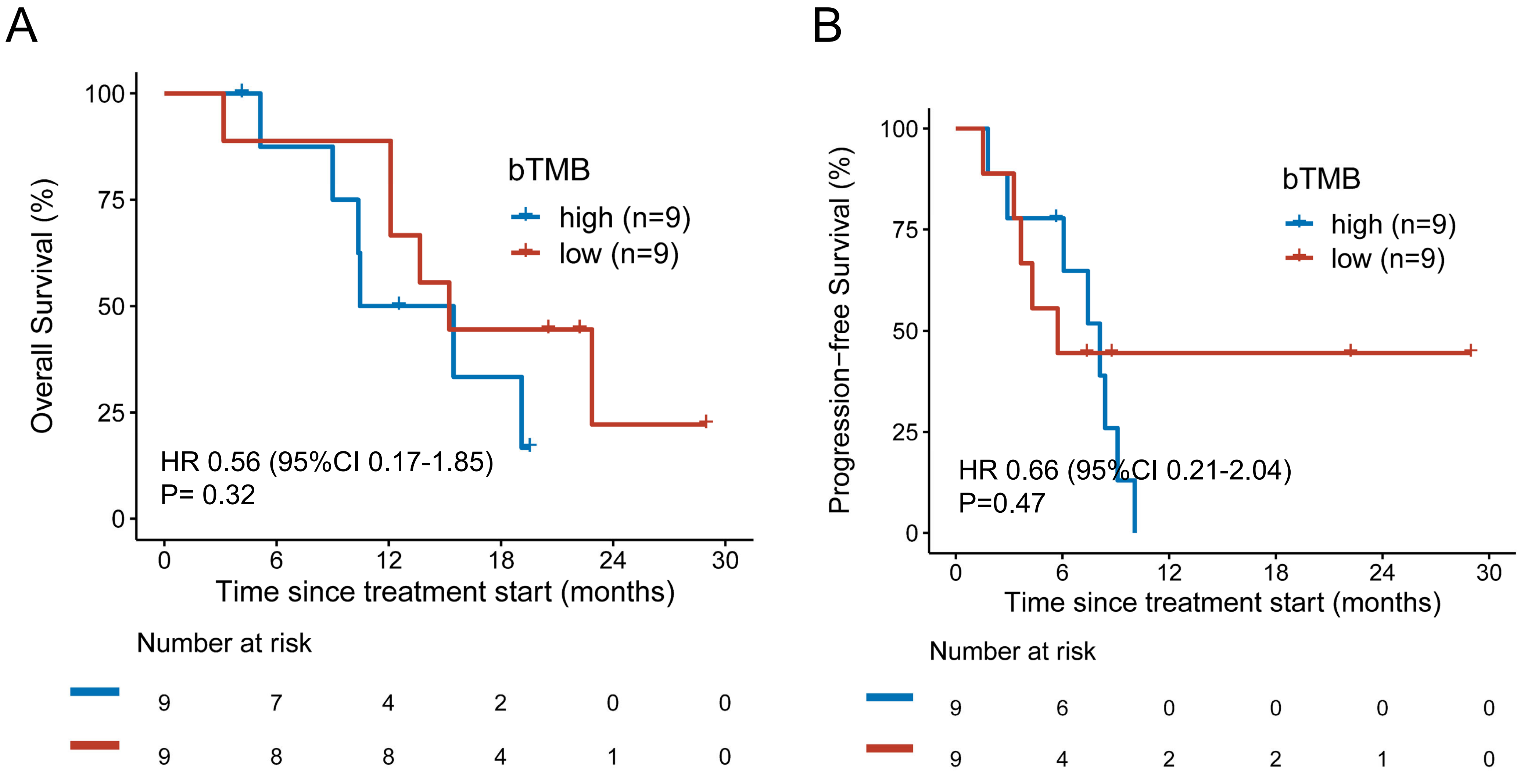
A
B
Overall Survival (%)
Progression−free Survival (%)
100
75
50
25
0
0
6
12
18
24
30
Time since treatment start (months)
bTMB
high (n=9)
low (n=9)
HR 0.56 (95%CI 0.17-1.85)
P= 0.32
HR 0.66 (95%CI 0.21-2.04)
P=0.47
Number at risk
9 7 4 2 0 0
9 8 8 4 1 0
9 6 0 0 0 0
9 4 2 2 1 0
